# Reply to the Letter of Terracini B. et al. “Comment on Piscitelli et al. Hospitalizations in Pediatric and Adult Patients for All Cancer Type in Italy: The EPIKIT Study under the E.U. COHEIRS Project on Environment and Health”. *Int. J. Environ. Res. Public Health* 2017, *14*, 495

**DOI:** 10.3390/ijerph14111291

**Published:** 2017-10-25

**Authors:** Prisco Piscitelli, Immacolata Marino, Andrea Falco, Matteo Rivezzi, Cosimo Neglia, Giulia Della Rosa, Giuseppe Militerno, Adriana Bonifacino, Gaetano Rivezzi, Roberto Romizi, Giuseppe Miserotti, Maurizio Montella, Fabrizio Bianchi, Alessandra Marinelli, Antonella De Donno, Giovanni De Filippis, Giuseppe Serravezza, Gianluca Di Tanna, Valerio Gennaro, Mario Ascolese, Alessandro Distante, Ernesto Burgio, Annamaria Colao

**Affiliations:** 1 Euro Mediterranean Scientific Bio-Medical Institute, ISBEM, Via Reali di Bulgaria, Mesagne, 72023 Brindisi, Italy; falco.and@gmail.com (A.F.); matteo.rivezzi@gmail.com (M.R.); neglia@isbem.it (C.N.); dellarosa.giulia@gmail.com (G.D.R.); distante@isbem.it (A.D.); 2 Department of Economics and Statistics and CSEF, University Federico II, 80131 Naples, Italy; miss.immamarino@gmail.com; 3 Local Health Authority ASL Napoli 3 South, 80100 Naples, Italy; g.militerno@gmail.com; 4 St. Andrea Hospital, La Sapienza University, 00185 Rome, Italy; adriana.bonif@gmail.com; 5 Division of Neonatology, St. Anna & St. Sebastiano Hospital, 81100 Caserta, Italy; isdecaserta@gmail.com; 6 Local Health Authority USL 8, 52100 Arezzo, Italy; isde@ats.it; 7 Local Health Authority USL Piacenza, 29121 Piacenza, Italy; giuseppe.miserotti@gmail.com; 8 IRCCS G. Pascale Foundation, National Cancer Institute, 80131 Naples, Italy; m.montella@istitutotumori.na.it; 9 National Research Council, CNR-IFC, 56121 Pisa, Italy; fabrizio.bianchi@ifc.cnr.it; 10 Department of Experimental Medicine, Second University of Naples (SUN), 80138 Naples, Italy; alessandra.marinelli@unina2.it; 11 Department of Biological and Environmental Sciences and Technologies (DISTEBA), University of Salento, 73100 Lecce, Italy; antonella.dedonno@unisalento.it; 12 Local Health Authority ASL LE, 73100 Lecce, Italy; giov.defilippis@gmail.com (G.D.F.); info@legatumorilecce.org (G.S.); 13 AMGEN International, 4057 Basel, Switzerland; gianluca.d@amgen.com; 14 IRCCS Policlinico San Martino IST, 16132 Genova, Italy; valerio.gennaro@hsanmartino.it; 15 Division of Pediatric Surgery, Salerno University Hospital “Ruggi D’Aragona”, 84100 Salerno, Italy; ascomar@tiscali.it; 16 European Cancer and Environment Research Institute (ECERI), 21004 Bruxelles, Belgium; erburg@libero.it; 17 Department of Clinical Medicine and Surgery, University Federico II, 80131 Naples, Italy; colao@unina.it

A letter to the IJERPH Editor was submitted by Terracini B. et al. as a comment to our latest paper “Hospitalizations in Pediatric and Adult Patients for all Cancer Type in Italy: The EPIKIT Study under the E.U. COHEIRS Project on Environment and Health” [1,2].

First of all, it is important to point out the added value represented by the wide panel of researchers from various institutions participating in the EPIKIT study group, which was assembled spontaneously as a result of the Europe for Citizens COHEIRS Project (Civic Observers for Health and Environment: Initiative of Responsibility and Sustainability) on precautionary principle.

In our opinion, Terracini et al. clearly assessed our paper from a perspective that is far removed from the aims of our research. Indeed, they made a comparison between Cancer Registry (CR) incidence data and the hospitalization figures provided in our work. Therefore, we were rather surprised by the statements contained in the comment letter, where Terracini and colleagues criticized us for having incorrectly estimated the “incidence” of cancer by using hospital discharge records. In no section of our manuscript did we state that our aim was to estimate incidence.

Since the “incipit” of the paper, we declared that “CRs are the gold standard methodology to collect epidemiological information on cancer incidence”, our aim being merely to make a first “attempt” to estimate the “burden” of cancer at regional and provincial level for the nation as a whole, including those areas that are still not covered by CRs. The aim of our study was clearly expressed as follows: “better understanding the consistency of social alarm that have spread in certain areas of the country concerning possible environmental threats to human health related to illegal activities leading to soil/air/water pollution” in order to be helpful “in explaining the widespread perception of higher incidence of tumors in pediatric population (0–19 years old) and adults belonging to those age groups (20–49 years old) generally excluded by official screening for cancer prevention”. At the same time, we limited our expectations by simply declaring that hospitalization data could be “used by decision makers in planning healthcare services to be offered at local level in the field of oncology”. All these statements are reported at the end of introduction section, where the rationale and the objective of the research are usually indicated. Thus, in our opinion, the assertion that we should have compared the hospitalization data resulting from our study with the incidence data produced by Cancer Registries is misleading.

Looking at this paper as a study providing incidence data is a misrepresentation that also explains the objections expressed by Terracini and colleagues concerning the supposed “overestimation of incident cases” in our paper. As a matter of fact, in the Methods section, we stated that “patients presenting the same identification number (treated anonymously) and the same major diagnosis were computed only one time”. On the contrary, this means that patients who were hospitalized with different major diagnosis codes (including different extensions of the same main code, i.e., 200.1 for the first hospital admission and 200.2 for the second) were computed in the analyses as well. This obviously generates an apparent “overestimation” if anyone looks at our results from the incorrect perspective of estimating “incident cases”. On the contrary, we clearly stated that “the incidence of all pediatric cancer cases in Italy is about 11,800 new cases per five years” in order to confirm that all pediatric hospitalizations analyzed in our paper had been suffered by about 11,800 new cancer patients per five years, as resulting from the Cancer Registries estimations. This confirms the reliability of CRs as the “gold standard” tool when searching for incidence data.

Despite the criticisms, we sincerely thank Professor Terracini and his colleagues, as their considerations give us valuable indications for improving our research from the perspective of assessing cancer-related hospitalization costs in Italy. Finally, we would like to take this opportunity to restate that, since the optimal tool for estimating cancer incidence is the population-based cancer registry, these should also be activated in those areas that are not yet covered by CRs, maybe giving priority to provinces with a high number of documented cancer-related hospital admissions.
